# Individualized Follow-up of Pregnant Women with Asymptomatic Autoimmune Thyroid Disease

**DOI:** 10.3390/ijms17010088

**Published:** 2016-01-12

**Authors:** Dana Stoian, Stelian Pantea, Madalin Margan, Bogdan Timar, Florin Borcan, Marius Craina, Mihaela Craciunescu

**Affiliations:** 1 Obstetrics Gynecology Department, “Victor Babes” University of Medicine and Pharmacy Timisoara, 2nd Eftimie Murgu Sq., Timisoara 300041, Romania; stoian.dana@umft.ro (D.S.); marganmm@gmail.com (M.M.); craina.marius@umft.ro (M.C.); 2 Surgery Department, “Victor Babes” University of Medicine and Pharmacy Timisoara, 2nd Eftimie Murgu Sq., Timisoara 300041, Romania; 3 Medical Informatics Department, “Victor Babes” University of Medicine and Pharmacy Timisoara, 2nd Eftimie Murgu Sq., Timisoara 300041, Romania; timar.bogdan@umft.ro; 4 Analytical Chemistry Department, “Victor Babes” University of Medicine and Pharmacy Timisoara, 2nd Eftimie Murgu Sq., Timisoara 300041, Romania; 5 Microbiology Department, “Victor Babes” University of Medicine and Pharmacy Timisoara, 2nd Eftimie Murgu Sq., Timisoara 300041, Romania; craciunescu.mihaela@umft.ro

**Keywords:** autoimmune thyroid disease, subclinical hypothyroidism, pregnancy, follow-up, supplemental therapy

## Abstract

Maternal hormones are essential for the normal fetal development during pregnancy. Autoimmune thyroid disease is a frequent pathology in our iodine replete region. The aim of this study is to evaluate the occurrence of subclinical hypothyroidism (SCH) in cases with known autoimmune thyroid disease, which were in a euthyroid state prior to pregnancy, and to assess the association between supplemental treatments administered and the outcome of the pregnancy. The study is a prospective interventional controlled study. The two cohorts comprise the interventional group, consisting of 109 pregnant women with known autoimmune asymptomatic thyroid disease, without any levothyroxine (LT4) treatment and an aged-matched control group, with an unknown thyroid disease. After the pregnancy, a monthly evaluation of TSH, FT3, and FT4 was performed. Offspring evaluation was made at birth time. 88.8% of the women developed SCH in the first four weeks of pregnancy. Average LT4 doses increased as the pregnancy progressed. The monthly adjustment was 12.5 or 25 μg. All SCH cases developed in the first trimester of pregnancy. There was no significant difference regarding the gestational week, weight, or length at birth between the interventional group and controls, when TSH values were in the optimal range, during the whole pregnancy. Premature birth was described in one case in the interventional group.

## 1. Introduction

In pregnancy, the maternal thyroid sustains the needs of the maternal-fetal unit. The fetal thyroid is functional only after month 5–6 of gestation [1].

Hypothyroidism in pregnancy is defined as the presence of an elevated TSH concentration during gestation. Elevations in serum TSH during pregnancy should be defined using pregnancy-specific reference ranges. Overt hypothyroidism (OH) is defined as an elevated TSH (>2.5 mIU/L) associated with a low FT4 concentration. Subclinical hypothyroidism represents a serum TSH between 2.5 and 10 mIU/L with a normal FT4 concentration [2].

A bicentric study [3] published in 2014 regarding the iodine status in Romania suggests that our country is iodine-sufficient in urban areas.

In iodine-replete areas, as in our region, the prevalence in the general population of high levels of thyroperoxidase antibodies (TPOAb) and antithyroglobulin antibodies (TgAb) is about 10%–20% of the healthy population [4]. High titers of TPOAb are detected in about half the pregnant women with SCH and in more than 80% of cases with OH [1,5,6,7].

Three hypotheses may cause the association of thyroid autoimmunity with pregnancy complications: firstly, thyroid autoantibodies may be considered as a marker of generalized autoimmune dysfunction in the body which itself has been known to be responsible for an increased pregnancy loss. Secondly, TPOAb euthyroid women before pregnancy are more prone to develop subclinical or overt hypothyroidism during pregnancy due to a hormonal imbalance in particular in the first trimester. Finally, thyroid autoimmunity is considered as one of the risk factors of infertility [8].

The insufficient concentration of thyroid hormones can cause: spontaneous abortion, preterm birth, preeclampsia, complications at birth, alteration of fetal brain development with decreased IQ (intelligence quotient), and cretinism [9]. SCH has been associated also with adverse events such as miscarriage, prematurity, gestational hypertension, placental abruption, as well as intellectual impairment in the offspring [10,11,12].

In addition, the presence of TPOAb is an independent risk factor for miscarriage, preterm delivery, perinatal death, and postpartum thyroid dysfunction [8]. The current recommendations [1] define SCH in pregnancy cases with TSH >2.5 mIU/L in first trimester, respectively over 3 mIU/L in the second and third trimester. Therefore, according to these recommendations, women that are considered euthyroid, before pregnancy, are reclassified as having subclinical hypothyroidism, in cases of TSH between 2.5 and 4 mIU/L.

The recommended treatment for maternal hypothyroidism consists of the oral administration of levothyroxine (LT4). Controversial opinions regarding the need of SCH treatment in pregnancy have existed in the past. In the last decade two guidelines, the 2007 Endocrine Society guideline and the 2011 ATA guidelines, state that women with positive antibodies and subclinical hypothyroidism should be treated with LT4. However, only a few studies on LT4 treatment in women with subclinical hypothyroidism during early pregnancy have been performed. The recommendation for universal treatment with FT4 is lacking because of insufficient data. The optimal dose of LT4 administered for SCH in pregnant women is still unknown [10,13,14]. However, the issue of euthyroid women, classified as asymptomatic autoimmune thyroid disease cases, is not specifically addressed in these guidelines. The recommendations are clear what to do in cases sub SCH and CH when pregnancy appears, but do not take into consideration the evolution of cases with normal TSH before pregnancy.

The aim of this study is to evaluate the occurrence of SCH in cases with known autoimmune thyroid disease, which were in a euthyroid state prior to pregnancy, and to assess the association between supplemental treatments administered, which have a positive impact on the outcome of the pregnancy.

## 2. Results

The moment of initial thyroid evaluation, after the pregnancy was confirmed, was between week 3.5 and week 5.5 (mean 3.98 ± 0.56 weeks) after the first day of the last menstruation.

The mean value of TSH before pregnancy was 2.67 mIU/L, significant lower as compared to the mean value of the first month of pregnancy, which was 4.62 mIU/L (*p* < 0.01). As presented in Table 1, a significant number of cases developed SCH shortly after the presence of pregnancy. The vast majority of cases developed SCH in the first two months of gestation: 88.99%, but the prevalence increased in the third month to 95.41%, in the fourth month to 97.24% and, in the fifth month, to 99.08%.

**Table 1:** Development and evaluation of SCH in women with pre-gestational euthyroid autoimmune thyroid disease.

Parameter	Pregestational	W5 ^1^	W10 ^1^	W15 ^1^	W20 ^1^	W24 ^1^	W28 ^1^	W32 ^1^	W36 ^1^	W40 ^1^	PP ^2^
TSH mIU/L	2.67 ± 0.28	4.62 ± 0.15	2.67 ± 0.11	2.10 ± 0.15	1.89 ± 0.07	1.79 ± 0.18	1.81 ± 0.09	2.02 ± 0.25	2.17 ± 0.23	2.30 ± 0.18	3.18 ± 0.76
Abnormal TSH (No)	0	97	+7	+2	+2	–	–	–	–	–	–
FT4 mIU/L	1.35 ± 0.67	1.25 ± 0.03	1.15 ± 0.01	0.97 ± 0.02	1.01 ± 0.01	1.25 ± 0.03	1.4 ± 0.04	1.67 ± 0.03	1.1 ± 0.01	1.7 ± 0.02	1.02 ± 0.01
Lt4 therapy % of cases	0	88.99	95.41	97.24	99.08	99.08	99.08	99.08	97.24	94.49	–
Average LT4 dose (mcg/day)	0	20.25	32.06	40.39	45.13	48.14	48.84	50.01	50.46	49.69	25.82

^1^ W5, W10····W40—week of gestation; ^2^ PP—postpartum evaluation.

A percent of 88.88% of the pregnant women with SCH received LT4 treatment very early in the pregnancy, more precisely after four weeks of gestation (G4). The mean TSH level in the first month of pregnancy was 4.62 mIU/L. After the first month of treatment with LT4 (a median dose of 20.25 μg/day), the serum TSH dropped from 4.62 to 2.65 mIU/L.

**Figure 1:**
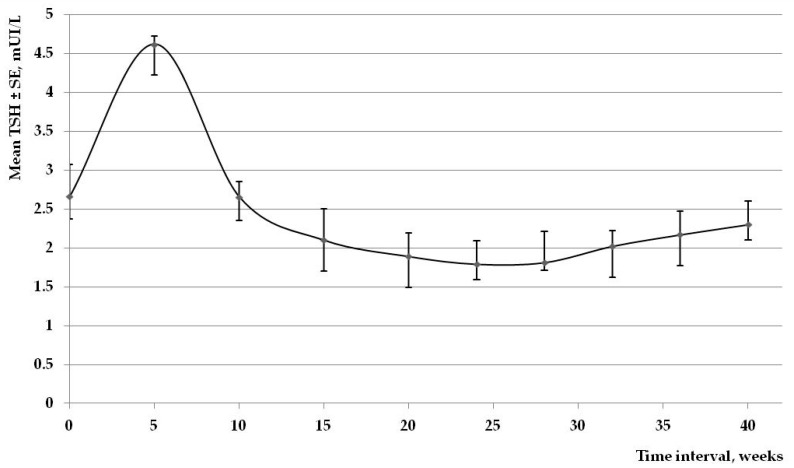
Mean TSH value during the pregnancies in the study group.

At three months of gestation the mean TSH value was situated at 2.10 mIU/L with a mean dose of LT4 situated at 40.93 μg/day. In the following months the mean TSH level had a fairly stable value until 40 weeks of gestation, when a small peak was noticed (2.30 mIU/L). At the same time, mean LT4 doses remained almost constant, gravitating around 50 μg/day (Figure 1).

### 2.1. LT4 Dosage in the SCH Pregnant Women

In Group 1 (patients with baseline = first endocrinological evaluation after conception, 0.5 < TSH < 2.5 mIU/L), at four weeks of gestation, none of the patients needed supplemental therapy. In the second month of gestation 7/12 patients needed supplemental therapy followed in the third month by another three cases. Only one patient did not need any supplemental therapy during the whole duration of pregnancy. Each patient received the optimal dose in accordance to the last administered dose and based on the last serum TSH value. In the second group (patients with baseline = first endocrinological evaluation after conception, 2.5 ≤ TSH< 4.5 mIU/L) the mean dose of LT4 at four weeks of gestation was 15.55 μg/day and the maximum mean dose was registered in the seventh month being 42.77 μg/day. All cases needed supplemental therapy starting with the first month of gestation. The same situation was seen in the third group (TSH ≥ 4.5 mIU/L at the first endocrinological evaluation after the conception). The levothyroxine mean daily dose was the greatest, starting with 33.33 μg/day in the first month, a dose that actually doubled to 60.62 μg/day at the end of pregnancy.

**Figure 2:**
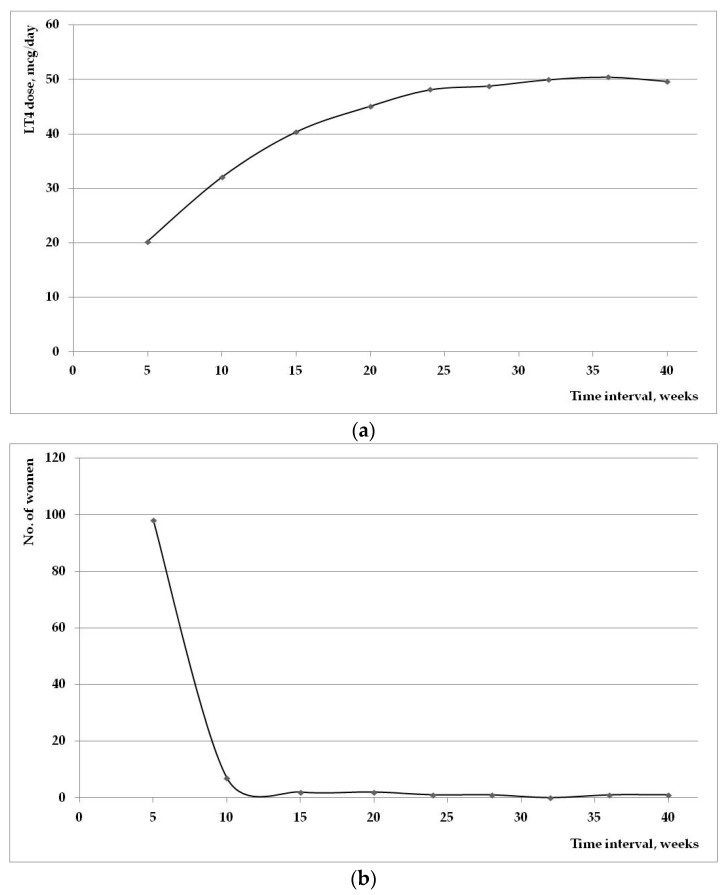
(**a**) Mean supplemental LT4 used dose during the evolution of pregnancy; (**b**) the number of cases that required initiation of LT4 therapy during the pregnancy.

The mean dose of LT4 doubled starting in W5 to final evaluation, in all three groups, having a linear increase in accordance with TSH values. Figure 2 represents the evolution of the mean used LT4 dose during the pregnancy (a); and the number of patients that required initiation of supplemental therapy (b). It is worth noticing that the majority of new introduced therapy was in the first trimester of pregnancy.

At baseline, there were no significant differences between the three groups of the SCH pregnant women regarding age, number of pregnancies and number of previous obstetrical events (early pregnancy losses, C sections, complication at delivery), or value of ATPO Ab. We have to mention that the assisted reproduced techniques (ART) cases were higher in Group 1. The limited number of cases, however, does not permit any conclusion regarding ART in asymptomatic autoimmune thyroid disease patients. Serum FT4 were similar in all three categories of cases. Only the value of TSH was different. As showed in Table 2, T test was used for the comparison of the three groups of cases. 

**Table 2:** Characteristics of study groups.

TSH at Baseline (mIU/L)	0.5< Group 1 <2.5	2.5 ≤ Group 2 <4.5	Group 3 ≥4.5	Total	Controls
No. of cases	12	46	51	109	109
Mean age (years)	33.34 ± 2.45	32.23 ± 3.11	31.85 ± 3.67	32.5 ± 3.13	32.3 ± 2.44
No of previous events (%)	17.14%	19.56%	18.51%	19.26%	18.32%
No. of ART cases	8	3	1	12	5
TSH W5 (mIU/L)	1.90 ± 0.52	3.65 ± 0.52	6.10 ± 1.12	4.62 ± 1.73	1.15 ± 1.78
FT4 W5 (ng/dL)	1.15 ± 0.45	1.32 ± 0.76	0.97 ± 0.04	1.25 ± 0.97	1.56 ± 0.67
ATPO Ab (UI/mL)	408.062 ± 238.52	326.83 ± 251.17	425.21 ± 298.88	398.15 ± 276.15	15.45 ± 4.56

**Figure 3:**
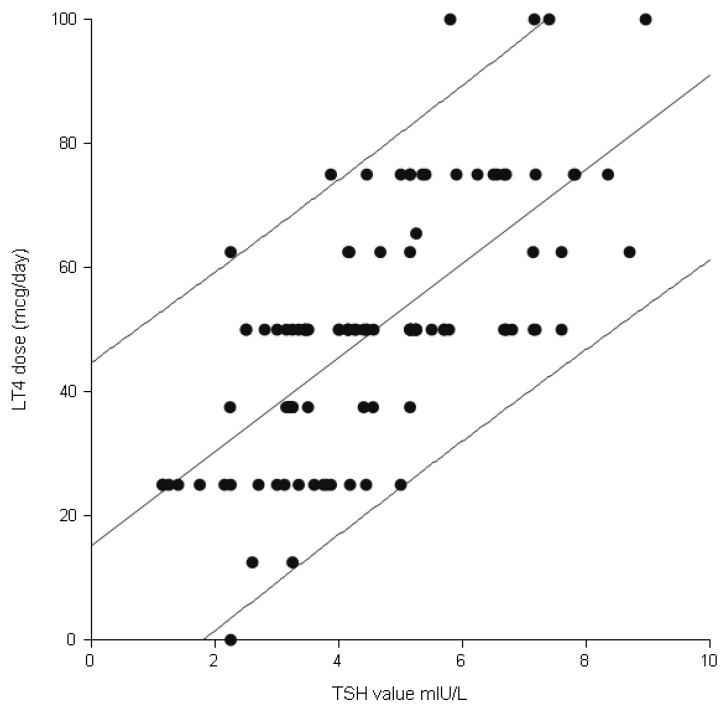
Linear regression of the final LT4 dose dependent on the initial TSH value, in cases with asymptomatic autoimmune thyroid disease.

After the initiation of therapy, as we see in Table 3, there was no difference in TSH and FT4 levels, during the whole pregnancy, but there were significant differences in the required LT4 dose in order to achieve normal TSH values. The higher the initial TSH value was, the higher the supplemental final doses were. There is a significant correlation between the initial changed TSH value, at the beginning of the pregnancy, and the final LT4 needed dose. Figure 3 demonstrates the excellent correlation of 0.69 (*R*^2^ = 0.75), (LCL 95% 0.534—UCL 95% 0.752), *p* = 0.001.

**Table 3:** Mean LT4 supplemental doses in all the three study subgroups, in all evaluation along the pregnancy.

Group LT4 mcg/day	Group 1 TSH < 2.5	Group 2 TSH 2.5–4.5	Group 3 TSH > 4.5	*p* (ANOVA)	*p*_(1_*vs*._2)_	*p*_(2_*vs*._3)_	*p*_(1_*vs*._3)_
W5	0	15.55 ± 5.37	29.16 ± 8.37	<0.001	0.001	0.001	0.002
W10	10.41 ± 10.43	24.72 ± 8.93	43.62 ± 11.09	<0.001	0.001	0.001	0.001
W15	17.70 ± 11.25	33.33 ± 11.78	51.96 ± 13.15	<0.001	0.002	0.001	0.002
W20	28.12 ± 14.22	38.05 ± 12.34	55.39 ± 13.38	<0.001	0.001	0.001	0.002
W24	30.20 ± 14.55	40.55 ± 13.73	59.06 ± 14.45	<0.001	0.001	0.001	0.001
W28	30.20 ± 14.55	41.38 ± 13.39	59.80 ± 14.90	<0.001	0.002	0.002	0.001
W32	30.20 ± 14.55	42.77 ± 14.55	61.03 ± 15.78	<0.001	0.001	0.002	0.001
W36	31.25 ± 16.42	42.50 ± 13.79	62.09 ± 16.14	<0.001	0.001	0.001	0.001
W40	31.25 ± 16.42	42.22 ± 14.26	60.62 ± 18.68	<0.001	0.001	0.001	0.002

*p* value was calculated using ANOVA test for the variance of the mean values between groups, respectively with Bonferroni *post hoc* test for the individual comparisons. W5 to W40 = weeks of gestation.

**Figure 4:**
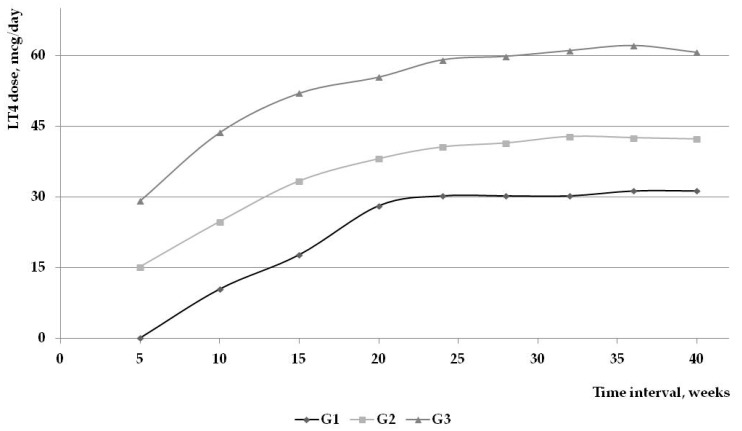
LT4 dose adjustments in the 3 subgroups.

As is shown in the Table 3, the higher increase in the supplemental dose was in the first trimester, in each group, the increment being similar. Worth mentioning is that a small number of cases (Group 1) had normal TSH values despite the presence of pregnancy, but this changed after the first two months of pregnancy. Figure 4 demonstrates that the main difference is the initial used dose: 0 mcg/day in Group 1, 15.55 ± 5.37 mcg/day in Group 2, and 29.16 ± 8.37 mcg/day in Group 3.

Additionally, the mean levothyroxine dose after the 24th week was stable in each analyzed groups.

The main difference in the three subgroups was the initial used dose, according to the TSH. As seen in Figure 4, the increment in the levothyroxine dose in the following month was similar.

After the first two months of pregnancy, the values of TSH remained stable during the entire duration of pregnancy. As seen in Figure 1, this individual monthly approach, with small increases in the LT4 doses, allows a stable control of maternal thyroid function.

### 2.2. Evolution in the Three Subgroups

**Figure 5:**
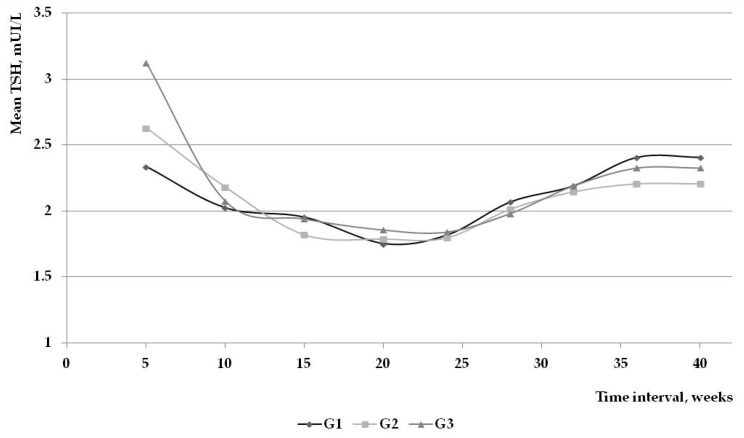
Evolution of mean TSH in the 3 subgroups.

After the initiation of the supplemental therapy, the TSH values in the first group, varied between 2.33 mIU/L (at four weeks of gestation) and 1.75 mIU/L (in fifth month), beginning to slowly increase until delivery. In the second group, TSH values ranged between 2.62 and 1.78 mIU/L and in the third group the values were between 3.12 and 1.84 mIU/L. Regardless of the study group, as presented in Figure 5, the vast majority of cases required therapy/therapy adjustment mainly in the first trimester of pregnancy.

As seen, there were no significant differences, among the three groups, neither in controlling TSH, nor in adjusting the LT4 dose. All patients had their TSH value correctly adjusted with an optimal dose of LT4, to obtain and maintain a TSH below 2.5 mIU/L in the first trimester, respectively below 3 mIU/L in the second and third trimester of pregnancy.

### 2.3. Proposed Algorithm

In cases with asymptomatic autoimmune thyroid disease (AITD), after the confirmation of pregnancy, TSH levels should be measured. In case of TSH below 2.5 mIU/L, no treatment should be recommended, only a monthly follow up. In cases of TSH higher than 2.5 mIU/L, levothyroxine treatment should be offered: 12.5 mcg if TSH is below 4.5 mIU/L and 25 in TSH if it is higher. Monthly increments of 12.5 mcg are recommended. After the 20th week, evaluations can be made every two to three months.

### 2.4. Pregnancy Outcome

From the 109 studied cases, 107 gave birth to offspring. Spontaneous miscarriage was observed in two cases in the first trimester of pregnancy.

In the 107 cases with delivery, one case with premature birth was recorded in week 36, and two cases of low birth weight for gestational weeks: 2250 g, gestational week 39, respectively 2450 g for gestational week 40, were also recorded. For the other 104 cases there were no differences regarding birth weight, birth length, APGAR score and time of birth as compared with controls. The data are presented in Table 4.

**Table 4:** Offspring characteristics in the study group compared with controls.

Parameter	APGAR SCORE	Week of Gestation	Birth Length (cm)	Birth Weight (kg)
Study group 107 cases	9.5 ± 0.7	39 ± 0.45	51.30 ± 8.70	3250 ± 703
Controls 109 cases	9.3 ± 0.6	38 ± 0.78	52.02 ± 9.20	3180 ± 653
*p*	0.135	0.455	0.245	0.134

### 2.5. Maternal Outcome

At two to three months after delivery, the maternal outcome was as follows:

Overt hypothyroidism was described in 65/109 cases (59.63%) with permanent need of levothyroxine replacement therapy. Postpartum thyroiditis with hyperthyroidism was observed in 8/109 cases (7.33%). After more than three months, all eight postpartum thyroiditis cases developed overt hypothyroidism. Only 36 cases (33.02%) returned to euthyroidism.

In the control group only one case of postpartum thyroiditis was recorded.

## 3. Discussion

The production of thyroid hormones by the pregnant women is the only source for the fetus, in the first and second semester of pregnancy, when neuronal development takes place [15]. Untreated SCH favors obstetrical complications, by two to three times [16], such as early pregnancy loss or preeclampsia. There is a well-documented effect of untreated hypothyroidism on the IQ, a reduction of 7 points on the scale, with additional motor, language and attention problems at 7–9 years of age [17].

Data evidences that LT4 treatment improves the course of pregnancy and can prevent obstetrical complications [11,18]. The data regarding the impact on neuro-intellectual development has not yet been proven [1].

In the current study, the number of females, with known autoimmune thyroid disease, with normal function previous to the pregnancy, who developed SCH in the first month of pregnancy was high, representing the majority of cases, 88.88% in the first month of pregnancy, which increased up to 99.08% at the end of the fourth month of pregnancy. Introduction of supplemental therapy achieved, in a maximum period of for weeks, normal TSH threshold values according to the Guideline recommendations [11]. The rapid compensation of TSH values, under correct LT4 supplement therapy, has been indicated also in the literature [1,10]. Regardless of the initial value of TSH, normalization was achieved in the majority of cases (93.75%) after the first four weeks of treatment.

After normalization of TSH, monthly increase in supplemental doses was similar, regardless of the initial TSH value. This readjustment of doses was required until the fifth month of pregnancy.

In our experience, the initial value of TSH is crucial in the recommendation of supplemental therapy, the higher the TSH value, the higher the initial supplementation dose. Choosing the right dose of supplemental therapy, TSH values normalize in the subsequent four weeks. The same conclusions are described in the literature; even higher levothyroxine doses than required have been recommended [19,20].

Other study groups recommended only an initial adjustment dose, with no further increments [18].

As a consequence of the supplemental therapy, the incidence of pregnancy loss was very low, 1.85% (two cases). The rate of miscarriages is lower than the prevalence described in the literature, in cases with positive ATPO Ab with normal TSH < 2.5 mIU/L—3.6%, respectively TSH = 2.5–5.5 mIU/L—6.1% [14,21].

The prevalence of premature birth in the intervention group, 0.93%, was also very low. The data from the literature is consistent, early levothyroxine in AITD cases reduces the risk of miscarriages [5,11,14,22].

Due to ethical reasons, we did not have a control group with autoimmune thyroid disease and suboptimal TSH value, where no intervention was offered.

The use of levothyroxine decreases the rate of obstetrical complications [21,23].

However, this rate is still higher than the rate described in the general population, of 0.5% [24]. This reference is the only current reference regarding abortion prevalence in our country.

An ideal TSH value before pregnancy allows a higher time interval till the introduction of supplemental therapy, with less possible addressability problems. Early levothyroxine treatment significantly reduces the risk of maternal hypothyroidism, in over 80% of cases in the first trimester, and mimics the normal maternal thyroid physiology [25].

The three obstetrical incidences observed during the study remain questionable whether they were directly related to the thyroid disease or the supplemental therapy.

A limitation of the study is that even if all the obstetrical events were recorded, because there was not an AITD placebo control group, only the number of events without the possibility of the calculation of risk reduction could be reported.

## 4. Materials and Methods

### 4.1. Patients and Methods

The study was performed in accordance with the ethical guidelines of the Helsinki Declaration, respecting local jurisdiction, and was approved by the Ethics Committee of the University of Medicine and Pharmacy, Timisoara, under the supervision of Prof Alexandra Enache, Approval No. 26, Approval date: 15 January 2014, Approval CECS 26/2014, the Institution where the study and the evaluations were performed. Signed informed consent was obtained previous to any medical intervention. Signed informed consent for publication of the results was also obtained.

### 4.2. Study Patients and Cohorts

The study is a retrospective controlled study. In the study we analyzed two cohorts: the autoimmune asymptomatic thyroid disease females who had become pregnant, *versus* a control group, age matched and also a historical control group. In the interventional study group, all 109 pregnant women with known autoimmune asymptomatic thyroid disease, with normal TSH before pregnancy, were enrolled.

Asymptomatic autoimmune thyroid disease is defined as: the increased titer of anti-thyroid antibodies (TPO Ab and/or Tg Ab) with or without changes on ultrasound: decreased thyroid volume, increased hypoecogeneity, pseudo-nodular disease and normal TSH values.

The euthyroid state in preconception, was without any supplemental LT4 therapy, and they became pregnant after the initial diagnosis. No supplemental therapy with thyroid hormones was used prior to the study. Both natural and assisted reproduced techniques (ART) obtained pregnancies were comprised in the study group.

Exclusion criteria: overt hypothyroidism at the moment of evaluation, previous use of levothyroxine therapy, previous subclinical/clinical hypothyroidism, previous thyroid surgery, any other coexisting systemic autoimmune disease, previous use of glucocorticoids or any other immunosuppressive therapy, secondary, or tertiary hypothyroidism and autoimmune thyroid disease (AITD) diagnostic made after the presence of pregnancy.

The pregnant women used supplements with folic acid or iron, if required. No iodine supplementation was used during the pregnancy in AITD cases.

The baseline evaluation was considered as the first endocrinological evaluation made after the positive diagnosis of pregnancy.

The median age in the study group was 32 years, ranging from 25 to 41 years old. Twin pregnancies were not encountered. 12 cases had assisted pregnancies: six insemination cases, with no follicular synthesis stimulation treatment; five cases were under Clomiphene citrate administration, and two cases had recombinant FSH follicular stimulation; the other 97 were natural pregnancies. The impact of recombinant FSH treatment on thyroid function was not evaluated due to the very small number of cases.

The study was performed between January 2014 and September 2015. The control, group comprised 109 age matched pregnant women, without known thyroid disease prior to the pregnancy, (normal anti thyroid antibodies titers, normal thyroid in ultrasound, normal TSH values in preconception and at the baseline). The control group was used for the evaluation of offspring parameters, compared with the offspring of the study group. The gestational age (weeks), length (cm), weight (gram), and APGAR score were recorded for each birth, both in the study group and in the control group.

Since the present study aims to develop a decision algorithm for the required and correct dosage of Levothyroxine treatment in cases with asymptomatic autoimmune thyroiditis, we divided the interventional cohort in three subgroups based on based TSH levels at baseline (first endocrinological evaluation after the pregnancy confirmation) and analyzed them accordingly: Group 1 (0.5 < TSH < 2.5 mIU/L) = 12 cases, Group 2 (2.5 ≤ TSH < 4.5 mIU/L) = 46 cases and Group 3 (TSH ≥ 4.5 mIU/L) = 51 cases.

### 4.3. Clinical and Biological Assessment

The diagnosis of autoimmune thyroid disease was established using the positive TPOAb, ranging from 87 to 1500 UI/L with or without any ultrasound changes. Women with previous hypothyroidism and supplemental treatment were not included in the study.

After the confirmation of pregnancy, SCH was defined as a serum TSH level higher than 2.5 mIU/L (first trimester) or 3 mIU/L (second and third trimesters), but lower than 10 mIU/L with normal serum free T4 (FT4). Overt hypothyroidism (OH) was defined as TSH level higher 2.5 mIU/L (first trimester) or 3 mIU/L (second and third trimester) with decreased FT4 concentration or TSH > 10 mIU/L, regardless of the FT4 levels. The definitions were used according to the ATA recommendations [2].

### 4.4. Evaluation

We evaluated the cases by monthly hormonal assays, thyroid ultrasound in the first and third semester.

Two experienced observers performed the thyroid evaluation with the same machine, HITACHI EUB 7500, with a multifrequency linear probe of 9–13 MHz.

The same laboratory performed hormonal assays.

The TSH assay used Chemiluminescent Microparticle Immunoassay, adult reference range of 0.53–4.20 mIU/L, with a detection limit of 0.0005 mIU/L.

The FT4 assay used Chemiluminescent Microparticle Immunoassay, adult reference range of 0.70–1.48 ng/dL, with a detection limit of 0.023 ng/dL.

Blood samples were taken in the morning in fasting status, before the use of any medication.

Outcome evaluation: obstetrical events were recorded, gestational age (weeks), length (cm), weight (gram) and Apgar score were recorded for each birth.

Maternal postpartum evaluation was made 8–10 weeks after birth given by means of clinical evaluation, TSH and FT4 assays, anti-thyroid antibodies measurement and thyroid ultrasound.

### 4.5. Algorithm

All patients were treated with LT4 as soon as hypothyroidism was confirmed during pregnancy, according to the current guideline recommendations [2]. The LT4 dose was considered adequate when a serum TSH of 2.5 mIU/L was reached in the first trimester or 3 mIU/L in the second and third trimesters. The treatment was individualized with either an increase of dose (in the vast majority of cases), or a decrease after the initial administration of LT4. The dose adjustment, either 12.5 or 25 μg, was made in accordance with the present TSH values, but also after studying the history of monthly evolution. LT4 dose was adjusted to maintain the serum TSH levels of pregnant women under control.

The gynecologist in our team recorded the evolution of the pregnancy. The outcome of pregnancy was also evaluated: gestational age of birth (weeks), length (cm), weight (kg), and APGAR score.

The incidence of miscarriage, premature delivery, gestational hypertension disease, gestational diabetes mellitus (GDM), placental abruption, or fetal distress was recorded during the study.

### 4.6. Statistical Analysis

Statistical analysis was performed using the NCSS version 9.0 for Apple (NCSS LLC, Kaysville, UT, USA). Data are presented as mean, SD, SE, 95% LCL and 95% UCL. P value was calculated using ANOVA test for the variance of the mean values between groups, respectively with Bonferroni *post hoc* test for the individual comparisons W5 to W40 = weeks of gestation. Spearman’s correlation was used regarding the study of the levothyroxine dose in respect to the TSH value.

## 5. Conclusions

The correct evaluation of asymptomatic autoimmune disease is required in cases of pregnancy occurrence. The prevalence of SCH in such cases is high, despite the euthyroid state before pregnancy. Due to this high prevalence of suboptimal TSH values, there should be a careful follow up of these “euthyroid apparent cases”. Ideally, preconception evaluation should be made in this special category of cases. An individualized treatment is recommended, with a careful follow-up of hypothyroid pregnant women and a systematic testing of thyroid function.

## Abbreviations

AITD: autoimmune thyroid disease
ART: assisted reproduced techniques
ATA: American Thyroid Association
ATPO Ab: antithyroperoxidase antibodies
FT3: free T3
FT4: free T4
GDM: gestational diabetes mellitus
IQ: intelligence quotient
LT4: levothyroxine
OH: overt hypothyroidism
PP: postpartum
SCH: subclinical hypothyroidism
TPO Ab: thyroperoxidase antibodies
TG Ab: thyroglobulin antibodies
TSH: thyroid-stimulating hormone
W: week of gestation
